# Robust Superhydrophobicity through Surface Defects from Laser Powder Bed Fusion Additive Manufacturing

**DOI:** 10.3390/biomimetics8080598

**Published:** 2023-12-12

**Authors:** Longxin Kan, Lei Zhang, Pengfei Wang, Qi Liu, Jihao Wang, Bin Su, Bo Song, Yusheng Shi

**Affiliations:** 1 State Key Laboratory of Material Processing and Die & Mould Technology, School of Materials Science and Engineering, Huazhong University of Science and Technology, Wuhan 430074, China; e0937509@u.nus.edu (L.K.); lzhan32@cityu.edu.hk (L.Z.); subin@hust.edu.cn (B.S.); shiyusheng@hust.edu.cn (Y.S.); 2 Department of Mechanical Engineering, National University of Singapore, Singapore 119077, Singapore; 3 Department of Mechanical Engineering, City University of Hong Kong, Tat Chee Avenue, Kowloon, Hong Kong 999077, China; 4 Advanced Materials and Energy Center, China Academy of Aerospace Science and Innovation, Beijing 100176, China; hvhe@163.com; 5 Science and Technology on Power Beam Processes Laboratory, Beijing Key Laboratory of High Power Beam Additive Manufacturing Technology and Equipment, Aeronautical Key Laboratory for Additive Manufacturing Technologies, AVIC Manufacturing Technology Institute, Beijing 100024, China; aliuqifrcn@sina.com (Q.L.); 18522586895@163.com (J.W.)

**Keywords:** 3D complex structures, additive manufacturing, surface defects, robust superhydrophobicity, superhydrophobic coating

## Abstract

The robustness of superhydrophobic objects conflicts with both the inevitable introduction of fragile micro/nanoscale surfaces and three-dimensional (3D) complex structures. The popular metal 3D printing technology can manufacture robust metal 3D complex components, but the hydrophily and mass surface defects restrict its diverse application. Herein, we proposed a strategy that takes the inherent ridges and grooves’ surface defects from laser powder bed fusion additive manufacturing (LPBF-AM), a metal 3D printing process, as storage spaces for hydrophobic silica (HS) nanoparticles to obtain superhydrophobic capacity and superior robustness. The HS nanoparticles stored in the grooves among the laser-melted tracks serve as the hydrophobic guests, while the ridges’ metal network provides the mechanical strength, leading to robust superhydrophobic objects with desired 3D structures. Moreover, HS nanoparticles coated on the LPBF-AM-printed surface can inhibit corrosion behavior caused by surface defects. It was found that LPBF-AM-printed objects with HS nanoparticles retained superior hydrophobicity after 150 abrasion cycles (~12.5 KPa) or 50 cycles (~37.5 KPa). Furthermore, LPBF-AM-printed ships with superhydrophobic coating maintained great water repellency even after 10,000 cycles of seawater swashing, preventing dynamic corrosion upon surfaces. Our proposed strategy, therefore, provides a low-cost, highly efficient, and robust superhydrophobic coating, which is applicable to metal 3D architectures toward corrosion-resistant requirements.

## 1. Introduction

Superhydrophobic matters, originally inspired by the lotus leaf [1], have attracted extensive attention, due to the features of self-cleaning, low friction, anti-corrosion, anti-icing, and anti-biofouling for applications in biotechnology, medicine, and heat transfer [2,3,4,5,6,7]. Superhydrophobicity is defined as when water droplets that contact these surfaces have large apparent contact angles (greater than 150 degrees) and small sliding angles (less than 10 degrees). This can be achieved by incorporating low surface energy and delicate micro/nanoscale surface roughness to maintain the substrate surface in the Cassie–Baxter state [8]. In this state, trapped air prevents extensive contact between the substrate and droplets, thereby preventing wetting. Moreover, micro/nanoscale structures exhibit unique physical and chemical properties due to their high specific surface area-to-volume ratio [9]. There are various methods to reduce the surface energy of solid surfaces, with two of the most widely employed approaches being the introduction or deposition of a layer of nanomaterials onto the solid surfaces and developing superhydrophobic surfaces, which is particularly effective in this regard, as it combines both low surface energy and nanolevel roughness on the solid surface [10]. This concept is well established, acknowledging that rough surfaces generally demonstrate superior hydrophobicity compared to flat surfaces [7].

Despite all these methodologies having been proven effective in conferring superhydrophobic properties to the substrate, surfaces tend to maintain their superhydrophobic capability at the cost of mechanical strength. This renders them fragile and highly susceptible to abrasion. Furthermore, three-dimensional objects with multidirectional curved outlines have a tendency to minimize the contact between liquid and solid surfaces. Consequently, a conflict arises between mechanical robustness and superhydrophobic capacity, particularly in the context of surfaces with intricate three-dimensional structures.

Various approaches attempt to balance the superhydrophobicity and their resistance to wear by strengthening the bonding layer or sacrificing the upper layers such as post-modification [9] and micro/nanostructuring throughout bulk objects [10,11]. Nevertheless, the anti-wetting capabilities can only remain for tens of mechanical frictions, and at present, only remain in effect for casted or molded structures with narrow geometrical complexity. A promising method using microstructural armor protection can yield robust superhydrophobic surfaces [12]. However, these materials have only been fabricated by the casting or molding method, with limited geometrical complexity.

Three-dimensional (3D) superhydrophobic objects can extend their applications from 2D surfaces to 3D components used across diverse industries. 3D printing is a disruptive technology that rapidly translates virtual 3D models into tangible 3D objects via digital assembly. Recently, a few studies have developed 3D printing to prepare superhydrophobic matters by directly printing the nano/microstructures to realize the surface roughness [13,14,15]. However, this direct printing strategy is small-scale, time-consuming, or easily deformable due to the balance between printing size and time. In addition, a developed methodology of combining a 3D direct laser printing system with polymerization-induced phase separation can leverage inherent nanoporosity to create superhydrophobic 3D objects, yet the robustness of the overall 3D structure is still limited [16].

Laser powder bed fusion additive manufacturing (LPBF-AM) has become a mainstream metal near-net forming technology and research hotspot in recent years, which provides a large number of solutions to the fabrication of robust and complex structures with unprecedented degrees of freedom [17,18,19,20,21]. However, the inhibition of LPBF-AM-printed metal objects with surface defects is a long-lasting challenge [22,23,24]. The inherent ridges and grooves’ surface defects existing in metal parts and surface hydrophilicity may accelerate the corrosion rate of the metal substrate, leading to equipment failure, reduction in component life, and even safety incidents [25,26]. Hence, it is urgent to endow superhydrophobicity to LPBF-AM-printed metal objects for improving the corrosion resistance of metals, especially in some extreme environments like high salinity and humidity [27,28].

**Figure 1:**
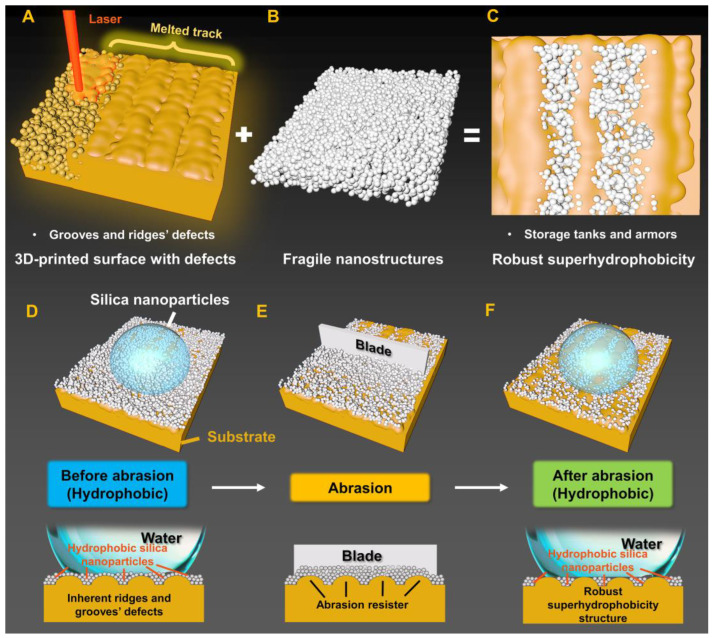
Design of the microstructure of robust superhydrophobicity surface. (**A**–**C**) The assembly process and intention of robust superhydrophobicity surface. (**A**) Schematics showing the metal 3D printed surface with grooves and ridges’ defects that exist in the melted tracks. (**B**) The fragile nanostructures. (**C**) Schematic showing the strategy for enhancing the mechanical stability of the superhydrophobic surface by housing water-repellent HS nanostructures within defects. The HS nanoparticles stored in the grooves among the laser-melted tracks serve as the hydrophobic guests while the ridges’ metal network provides the mechanical strength. (**D**–**F**) Schematics showing the mechanism of metal 3D printed robust superhydrophobicity surface developed by defects. (**D**) The HS nanoparticles provide water repellency. (**E**) Protection afforded by the topology of grooves and ridges’ microstructures. Abrasion objects that are larger than the grooves are blocked by the ridges’ microstructure. (**F**) The superhydrophobicity is well retained after abrasion.

Here, we propose a strategy that takes the inherent ridges and grooves’ surface defects from LPBF-AM as storage spaces for coating hydrophobic silica (HS) nanoparticles to obtain superhydrophobic capacity and superior robustness (Figure 1). Due to the inherent process characteristics of the LPBF-AM process, laser melting of metal powder produces melting paths, which has the microstructure of grooves and ridges (Figure 1A). The HS grains stored in the grooves among the laser-melted tracks serve as the hydrophobic guests (Figure 1B), while the ridges’ metal network provides the mechanical strength (Figure 1C). The surface of LPBF-AM-printed objects covered by HS nanoparticles act out good water repellency (Figure 1D). The grooves and ridges’ microstructure can prevent the HS nanoparticles from being removed by abradants that are larger than the grooves’ size (Figure 1E). After abrasion, HS particles can still remain in the grooves due to the distributed robust ridge structures (Figure 1F). Further, we systematically investigate the process-structure-properties relationship among LPBF-AM-printing parameters, surface structures, mechanical strength, and superhydrophobic capacity of metal 3D printed objects. Finally, we present that due to the defects covered by HS nanoparticles, the metal 3D printed objects possess excellent corrosion resistance under the impact and alkaline environment of seawater.

The above strategy for manufacturing superhydrophobic surfaces contributes at least two advantages. The former is that the coated superhydrophobic nanoparticles are good at water repellency and could be stored well in the grooves under the robust ridges’ metal network. The surfaces of LPBF-AM-printed components such as corrosion-resistant film can also be superhydrophobic objects, which possess complex and porous structures and exhibit a good resistance to wear which maintains the superhydrophobicity. The latter is the rapid and precise fabrication of digitally designed 3D metal products with high complexity that are beyond the traditional manufacturing approaches.

## 2. Materials and Methods

### 2.1. Materials

Hydrophobic silica (AEROSIL R202) was provided by Shanghai Kaiyin Chemical Co., Ltd. (Shanghai, China). Stainless powder (316L), NiTi powder, CoCr powder, and Ti powder were provided by Nantong ZZT SRIM Additive Manufacturing Co., Ltd. (Nantong, China). Polylactic acid (PLA) was purchased and used as received.

### 2.2. Fabrication of Metal Substrate

All stainless steel (316L) samples were fabricated using the selective laser melting system (HK M125, Huake 3D Technology Co., Ltd., Wuhan, China). The process parameters were as follows: laser power 250~350 W, scanning speed 550~750 mm/s, scanning pitch 0.1 mm, layer thickness 0.03 mm, spot diameter 0.1 mm.

All the NiTi, CoCr, and Ti samples were fabricated using the selective laser melting system (Fastform FF-M140, Meiguang Fastform Technology Co., Ltd., Dalian, China). The process parameters were as follows:

For NiTi samples: laser power 110 W, scanning speed 500 mm/s, hatching distance 0.1 mm, layer thickness 0.03 mm.

For CoCr samples: laser power 155 W, scanning speed 950 mm/s, hatching distance 0.1 mm, layer thickness 0.03 mm.

For Ti samples: laser power 185 W, scanning speed 850 mm/s, hatching distance 0.1 mm, layer thickness 0.03 mm.

### 2.3. Fabrication of 9 Stainless Steel (316L) Samples with Different Energy Densities

It is widely accepted that the process parameters have a significant influence on the topography of selected laser melting samples. Previous studies have indicated that some typical topographies with different roughness, such as holes, discrete weld beads, continuous weld beads, and spatter particles can be found with a variation in laser power and scanning speed.

In this study, we set the laser power at 250~350 W and the scanning speed at 550~750 mm/s according to the process window of stainless steel (316L) on the HK M125 platform (Huake 3D Technology Co., Ltd., Wuhan, China). The microstructure and roughness of samples were tested, and the mechanical stability was measured.

### 2.4. Fabrication of Superhydrophobic Coating

Hydrophobic silica nanoparticles were dispersed in ethanol at a weight ratio of 1:10 and stirred for 3 min until no obvious particles were observable on the surface of the ethanol. The resulting solution was then meticulously applied to the metal samples using a dropper, ensuring comprehensive coverage of the entire surface for a more uniform coating. The nanometer particle size and robust wettability of ethanol facilitated the effective penetration of the nanoparticles and ethanol mixture into all the surface’s holes and grooves.

Following the application, the treated samples were carefully placed in a constant temperature box set at 60 °C for a duration of 1 h to promote thorough drying. Throughout this meticulous process, the hydrophobic silica nanoparticles underwent deposition onto the metal surface, seamlessly filling the holes and grooves as the ethanol evaporated. The result is a thin and evenly distributed coating of nanoparticles, significantly enhancing the protective properties of the metal surface.

### 2.5. Fabrication of Experimental Apparatus

Most of the experimental apparatus used in this study were prepared on fused deposition modeling (FDM) equipment (Ultimaker 3, Ultimaker Co., Ltd., Geldermalsen, The Netherlands). These included the support frames of the real-time force measurement platform, the support frames of the ship-like samples, and the wave-triggered frames. To ensure the required strength, tough PLA was used, the process parameters of which were as follows: print speed 70 mm/s, layer height 0.1 mm, wall thickness 1 mm, infill density 20%, printing temperature 200 °C, and build plate temperature 60 °C.

### 2.6. Microstructure and Roughness

A white light interferometer (High Precision Surface Profiler) system (NewView 8300, Zygo Co., Ltd., Middlefield, CT, USA) was used to measure the surface roughness of samples. An electron probe microanalyzer (Quanta650 FEG, FEI Co., Ltd., Hillsboro, OR, USA) was employed for microstructural examination and element mapping. An electron probe microanalyzer (EPMA-8050G, SHIMADZU Co., Ltd., Kyoto, Japan) was employed for the microstructural examination of powders and particles.

A Mastersizer 3000 (Malvern Panalytical Co., Ltd., Marvin, UK) was employed for the particle size tests.

### 2.7. Linear Abrasion Test

Linear abrasion testing was applied to evaluate the mechanical robustness of samples with different laser powers and scanning speeds. This test was carried out utilizing SiC sandpaper (1200 grit). The lengths, widths, and heights of the tested samples were 20 mm, 20 mm, and 5 mm, respectively, the load applied was 200 g, and the abrasion distance of one cycle was 10 cm. The weight was stuck to the top of the samples, and the sample was pushed from one side to the other side.

### 2.8. Wetting Characterization

The measurement of static contact angles and sliding angles was carried out in the static mode and the rolling mode using a contact angle meter (SDC-350, SINDIN, Dongguan, China). Droplets (4 μL) were dispensed onto the test surface. Images of droplets were captured using a horizontal microscope equipped with an angle meter. The static contact angle was calculated using ImageJ software (ImageJ1) to obtain accurate values.

The platform was then rotated at a speed of 1°/s and stopped when droplets (4 μL) began to roll. The acquisition frequency was set at 5 frames/s to enable the rolling moment and sliding angle to be accurately captured. At least 5 different locations for each sample were measured to reduce error.

### 2.9. Water Droplet Bouncing Test

The water bouncing process on different surfaces was recorded on a MEMRECAM HX-7s high-speed camera system (ST-857, NAC Image Technology Inc., Tokyo, Japan) controlled by MEMRECAM HXLINK (SP-642). The acquisition frequency was 2000 frames/s.

## 3. Results and Discussion

Laser processing parameters determine the surface morphology of LPBF-AM-printed samples. Before characterizing the water repellency and wear resistance of the superhydrophobic surface, we optimized the laser processing parameters. A series of LPBF-AM-printed stainless steel (316L) samples were prepared using diverse laser power and scanning speeds (Figures S1–S3), of which the sample fabricated with a laser power of 350 W and scanning speed of 550 mm/s (marked as #3 in the Supplementary Materials) shows a ridge-and-groove staggered structure with a roughness (*S_a_*) of ~10 μm (see in Table S1). The *S_a_* represents the average of the absolute values of the height differences between points relative to the average surface of the surface and is generally used to evaluate surface roughness, which could be calculated by Equation (1).

(1) $$S_{a}=\frac{1}{NM}\sum_{i=1}^{N}\sum_{j=1}^{M}\left|Z_{ij}\right|$$

where *Z* is the distance from the point on the contour of the object surface area to the reference plane, and *M* and *N* are the sampling points in two mutually perpendicular directions in the evaluation area, respectively.

**Figure 2:**
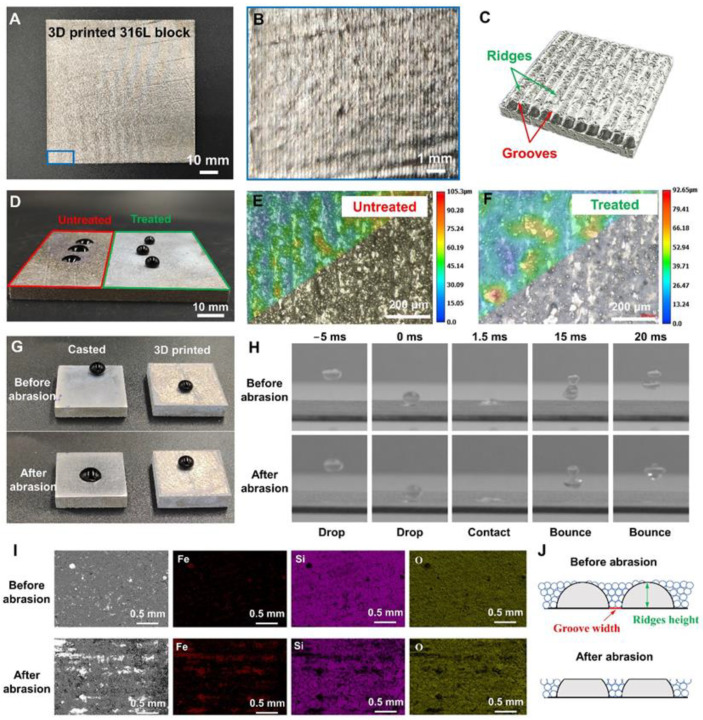
Metal 3D printed superhydrophobic surface. (**A**) Optical image of LPBF-AM-printed 316L block under optimized laser parameters. (**B**) The localized surface morphology in (A) showing grooves and ridges’ defects. (**C**) Schematic diagram of melted tracks for distinguishing grooves and ridges’ microstructures. (**D**) The water repellent comparison between the untreated surface without HS grains and the treated surface with HS grains. (**E**,**F**) The super depth-of-field images and optical micrograph show the typical morphologies of (**E**) untreated and (**F**) treated LPBF-AM-printed surfaces. (**G**) The optical images for the water repellent comparison of the casted surface and the LPBF-AM-printed surface before abrasion and after abrasion (200 g, 10 cycles). (**H**) Time-lapse images of water droplets bouncing on the LPBF-AM-printed superhydrophobic surfaces before abrasion and after abrasion (200 g, 10 cycles). (**I**) Typical microstructures of LPBF-AM-printed superhydrophobic metal system before and after abrasion. The uniform and thick superhydrophobic coating could be detected before abrasion in SEM images and element mapping. The superhydrophobic coating was still kept in the grooves after abrasion in SEM images and element mapping. (**J**) Cross-section schematic showing the action of grooves and ridges’ microstructures for robust superhydrophobicity.

This kind of surface roughness is suitable for providing the storage space for reserving the hydrophobic grains, leading to further robust superhydrophobic engineering. Figure 2A,B shows optical images of an optimized sample, including the typical ridge-and-groove staggered structures of SLM parts. A schematic diagram of melted tracks for distinguishing grooves and ridges’ microstructures is shown in Figure 2C. The ridges are the laser melting tracks, while the positions between the melted tracks are grooves. Figure 2D shows the transition from hydrophilic (contact angle ~77°) to superhydrophobic (contact angle ~152°) of specific parts after treatment. The super depth-of-field images and optical micrograph in Figure 2E,F shows the typical structures of untreated and treated SLM surfaces. Many uniform ridges and grooves can be observed. HS grains are coated in the surface’s grooves and ridges caused by the LPBF-AM-printed process, which presents better surface planeness than the untreated LPBF-AM-printed surface.

To demonstrate the unique adaptation of LPBF-AM-printed objects for this robust superhydrophobic strategy, casted samples with the same hydrophobic treatment process were used as the control experiment. As exhibited in Figure 2G, the superhydrophobic coating of casted parts (with smooth surface) was only destroyed after the abrasion of 10 cycles under 200 g (500 Pa) since the HS nanoparticles could hardly be stored during the abrasion process, while the LPBF-AM-printed parts still maintain their superhydrophobicity. Time-lapse images both present the water droplets bouncing on the LPBF-AM-printed superhydrophobic surfaces before abrasion and after abrasion (200 g, 10 cycles), showing good water repellency, as shown in Figure 2H. The LPBF-AM-printed sample can still maintain good water repellency after abrasion due to the storage of HS nanoparticles in the groove on the surface of the LPBF-AM-printed sample, which can be found in the elemental mapping top SEM observations before and after abrasion (Figure 2I). Before abrasion, it can be found that the height of HS nanoparticles coated on the surface of the LPBF-AM-printed sample is higher than that of the grooves, so that it is difficult to find the Fe element. After abrasion, the surficial HS nanoparticles and convex ridges are partly consumed, presenting a situation where the ridges and grooves intersect and coexist (Figure 2J). The HS nanoparticles disperse uniformly on the ridge-and-groove staggered structure, resulting in considerable roughness and low surface energy. This is one of the advantages of the LPBF-AM printing strategy.

Above, we show the storability of HS nanoparticles on ridges and grooves of the surface defects of the LPBF-AM-printed sample, as well as its robust superhydrophobicity. The correlation between laser processing parameters and their superhydrophobic capacity needs to be further clarified. The laser energy density (LED) is an important parameter on the performance of the printed samples in the LPBF-AM 3D printing technology, which could be calculated using Equation (2), and *P*, *v*, *h*, and *H* denote the laser power, scanning speed, hatching distance, and layer thickness, respectively.

(2) $$LED=\frac{P}{v\times h\times H}$$

**Figure 3:**
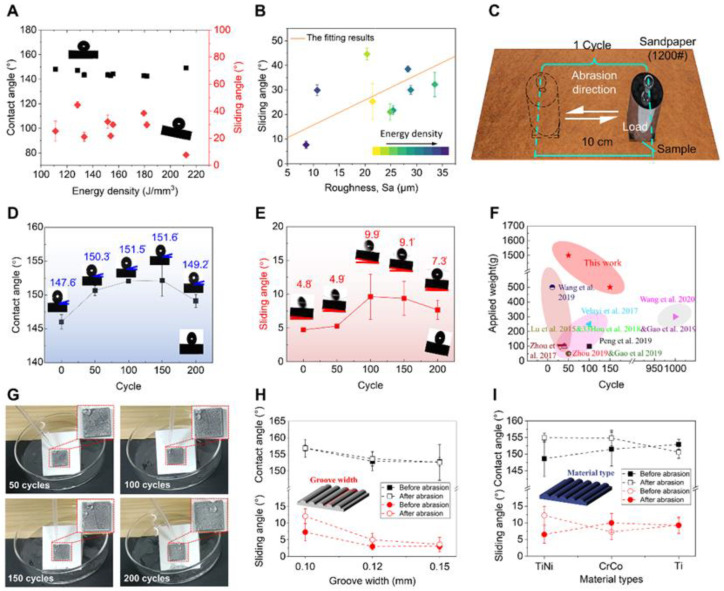
The process-structure-properties relationship among LPBF-AM process parameters, surface structures, mechanical strength, and superhydrophobic capacity of metal 3D printed objects. (**A**) The plot of contact angles and sliding angles versus energy density of LPBF-AM-printed surface. (**B**) The relationship between the sliding angle, roughness, and energy density. (**C**) Abrasion test. The influence of linear abrasion cycle numbers under 200 g loads on (**D**) contact angle and (**E**) sliding angle of LPBF-AM-printed surface. (**F**) Comparison of mechanical stability in this study and those currently reported for other current approaches [12,29,30,31,32,33,34,35,36,37]. (**G**) Optical images of abrasion test under 1500 g. (**H**) The contact angle and sliding angle of samples with different widths of grooves with 0.10 mm, 0.12 mm, and 0.15 mm. (**I**) The material types of LPBF-AM-printed surface versus the contact angle and sliding angle.

Figure 3A shows that the contact angle of LPBF-AM-printed samples is nearly 150°, ignoring the influence of laser energy density, but the sliding angle approximation is negatively correlated with the energy density. This is due to the difference in surface roughness of LPBF-AM-printed samples under different laser energy densities. A positive correlation between roughness and sliding angle was observed, although there is no obvious rule between laser energy density and roughness (Figure 3B). The LPBF-AM-printed sample with the lowest sliding angle and highest energy density is considered the best sample with the robust superhydrophobic capacity, which is also the sample used for the above water repellency characterization and used for further study.

To evaluate the mechanical robustness of the superhydrophobic LPBF-AM-printed sample, abrasion testing was applied to the sample and then its contact angles and sliding angles were measured. This abrasion test was carried out utilizing silicon carbide sandpaper (1200 grit). Figure 3C is a schematic diagram of the sandpaper abrasion test; the load applied was 200 g, 500 g, 1000 g, and 1500 g (500 Pa, 1.25 KPa, 2.5 KPa, 3.75 KPa) and the abrasion distance in one cycle was 10 cm. The length, width, and height of the tested sample were 20 mm, 20 mm, and 5 mm, respectively. The sandpaper abrasion test processes are shown in Movie S1. As exhibited in Figure 3D,E, the contact angle (droplet size 4 μL) and sliding angle (droplet size 4 μL, and rotation speed 1°/s) of LPBF-AM-printed samples vary with the number of abrasion cycles under 200 g loads. The contact angle is still about 150° and the sliding angle is still less than 10° during the 0 to 200 abrasion cycles. Further abrasion tests showed that the sample maintained the superhydrophobicity after 150 cycles and 50 cycles with 12.5 KPa and 37.5 KPa, respectively (Figure 3F and Figure S4, and Movies S2–S4). This result is much higher than that of currently reported data. And even shows non-wettability after 200 cycles with 12.5 KPa, 25 KPa, and 37.5 KPa (Figure 3G).

In addition, we changed the samples by designing different groove widths and material types (Figure 3H,I). As shown in Figure 3H, the contact angle and sliding angle vary with the width of the grooves. The sample with a groove width of 0.12 mm shows the smallest sliding angle (less than 5° during the entire abrasion process), while the sample with 0.10 mm grooves has the largest contact angle (more than 155° during the entire abrasion process). This is attributed to the larger groove width having more HS nanoparticles, which makes the surface of the LPBF-AM-printed sample more water repellent. However, excessive grooves will reduce the abrasion-resistance performance of ridges. The LPBF-AM-printed samples with appropriate grooves have a smaller sliding angle reduction and higher robustness after abrasion, which is also due to it having more of its own HS nanoparticles and a considerable number of ridges. Moreover, compared with the samples with grooves of 0.10 mm and 0.15 mm, the LPBF-AM-printed samples with grooves of 0.12 mm presented a lower surface roughness before and after abrasion (Figure S5). Although LPBF-AM-printed samples with different groove widths have different contact angles and sliding angles, they exhibit robust superhydrophobic properties before and after abrasion (Figure S6, and Movies S5 and S6).

To demonstrate the wide adaptation of this strategy of LPBF-AM-printed robust superhydrophobic surfaces, diverse material species were utilized (Figure 3I). Different samples (NiTi, CoCr, Ti) were fabricated using the LPBF-AM process (the microstructure and distribution of the particle size of the powders used in this work are shown in Figure S7). The sliding angle of LPBF-AM-printed samples with the CoCr material shows an obvious increase after abrasion, while the Ti-based sample remains unchanged. On the other hand, the contact angles of CoCr and TiNi materials are significantly increased while the Ti material is slightly decreased, and yet shows >150° superhydrophobic states. This divergence in superhydrophobic properties may be attributed to the printability, surface quality, and surface hardness of the different materials (Figure S8). These tests indicate that these LPBF-AM-printed sample surfaces with different metal materials could gain the non-wetting property after treatment with HS nanoparticles and keep this property after our mixed abrasion cycles (Figure S9, and Movies S7 and S8). In addition, it was found that LPBF-AM-printed samples with different grooves and different materials all remained water repellency even after undergoing different abrasion tests, including the knife cutting test, file underside and flank grinding test, as well as the sandpaper friction test, illustrating their robust superhydrophobic stability (Figure S10). This also proves the wide applicability of our strategy.

**Figure 4:**
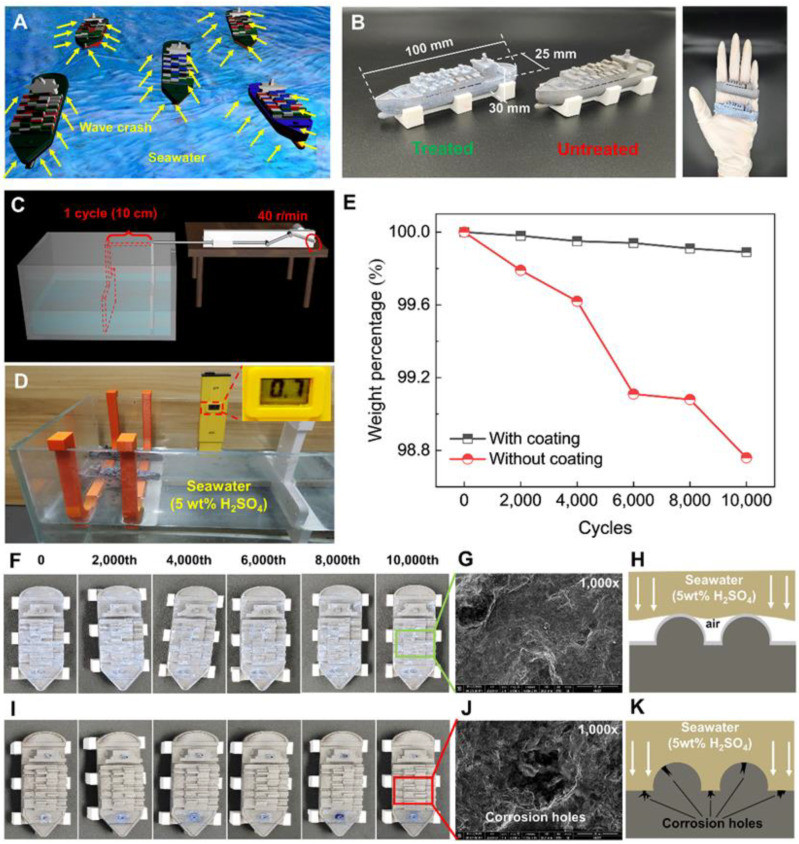
LPBF-printed corrosion-resistant ship body. (**A**) Schematic diagram showing the scouring of waves encountered by ships at sea. (**B**) LPBF-printed treated and untreated ship body samples with length, width, and height of 100 mm, 25 mm, and 30 mm. (**C**) Schematic diagram showing stroke and rotation speed of homemade wave trigger machine. (**D**) The liquid in the homemade wave trigger machine was immersed in a mixture of seawater and sulfuric acid (5 wt%) with a PH value of 0.7. (**E**) Residual weight percentage of LPBF-AM-printed ship-like samples with and without coating. Optical images of (**F**) treated and (**I**) untreated samples during 0–10,000 cycles. SEM images showing (**G**) superhydrophobic coating and (**J**) corrosion holes, and a schematic diagram of the impact of seawater on (**H**) treated sample and (**K**) untreated sample.

Utilizing the superhydrophobic coating to prevent corrosion is common, and many static superhydrophobic engineering strategies have been reported [32,38,39]. However, the ocean is ‘alive’ and ‘kinetic’, and the waves and the corrosiveness of seawater are significant challenges for ships at sea (Figure 4A). Millions and even billions of dollars of losses are caused by corrosion on ships every year [40,41]. Hence, we demonstrated the possibilities to protect ships from hydro-corrosion of seawater. We designed and LPBF-AM-printed two metal “ships” with typical ridge-and-groove staggered structures on their surfaces. As shown in Figure 4B, the length, width, and height of the ships are 100 mm, 25 mm, and 30 mm, respectively. As Figure 4C,D indicates, to enhance the speed of corrosion, the samples were immersed in a mixture of seawater (gathered from Weihai, Shandong province, China) and sulfuric acid (5 wt%) with an initial PH value of 0.7. The waves were triggered by a reciprocating crank slider mechanism with a stroke of 10 cm and a speed of 40 r/min (Figure 4C; The testing process is shown in Movie S9).

The weight of the samples was measured on a periodic basis. Figure 4E shows the percentage change in the residual weight of coated and uncoated LPBF-AM-printed samples. LPBF-AM-printed samples with superhydrophobic coating have less mass loss than that without superhydrophobic coating, showing that the superhydrophobic coating plays a vital role in protecting the samples from corrosion. The percentage of weight loss of samples with the protective layer and without the protective layer is about 0.1% and 1.3%, respectively, during the 10,000 cycles wave scouring test. Optical (Figure 4F,I) and SEM images (Figure 4G,J) reveal the reasons why the coating is effective in reducing corrosion (further SEM images are shown in Figure S11). It was found that the treated samples still kept superhydrophobicity and the spread of the intact coating can be observed in both optical images and SEM images, although the coating became thinner after 10,000 cycles. While the untreated sample was heavily corroded, a great number of newly emerged microcorrosion holes can be observed after 10,000 cycles. Figure 4H,K reveals the impact of seawater on treated and untreated samples. It is obvious that our LPBF-AM-printed robust superhydrophobic metal system shows great potential for the protection of metal substrates.

The inherent ridge-and-groove staggered structures of LPBF-AM-printed metallic objects accelerate metal corrosion. However, the above structures are also potential anti-abrasion structures and storage tanks for HS nanoparticles. The combination of LPBF-AM-printed surface defects and HS nanoparticles aids the construction of a robust superhydrophobic metal system to avoid metal substrate corrosion. Anti-corrosion tests of ship-like samples showed that our work has the potential to protect ships from waves and corrosion caused by seawater. This strategy has a number of attractive advantages, including its low cost, high efficiency, and a wide choice of materials. We believe that this robust superhydrophobic metal system design provides a novel solution that would boost the development of additive manufacturing and functional coatings.

However, our work still has some limitations. For example, there are no physical or chemical connections between the superhydrophobic coatings and the metal substrates. Hence, this strategy still has the risk of failure under heavy loads or harsh environments. This is expected to be solved by embedding superhydrophobic powders into metal substrates under high temperature and pressure, or using chemical methods to build intermediate layers or introducing van der Waals forces. In addition, the inherent ridge-and-groove staggered structures only exist on some specific surfaces (for example, the top surfaces) because parts are fabricated layer-by-layer, and only the top surface, for example, shows melt tracks. There are no such structures on the side or bottom of the parts. With the development of metal 3D printing technologies, it could be expected that all the 3D printed metal surfaces exhibit the same surface structures. Hence, our proposed strategy shows the prospect of constructing a low-cost and easy-access superhydrophobic coating with high efficiency. In addition, the stability and the universality of the LPBF-AM-printed robust superhydrophobic metal system could be improved by solving the limitations above.

## 4. Conclusions

We proposed a strategy that takes the inherent ridges and grooves’ surface defects from the laser powder bed fusion additive manufacturing process to obtain superhydrophobic capacity and superior robustness. The HS nanoparticles stored in the grooves among the laser-melted tracks serve as the hydrophobic guests while the ridges’ metal network provides the mechanical strength, leading to robust superhydrophobic objects with desired 3D structures. In addition, we have confirmed the versatility of this strategy, which can exert robust superhydrophobicity under different metal materials, different abrasion patterns, and different groove width designs. Abrasion resistance results show LPBF-AM-printed parts remained superior hydrophobicity after 150 abrasion cycles (~12.5 KPa) or 50 cycles (~37.5 KPa), which surpasses most reported results. Moreover, HS nanoparticles coated on the LPBF-AM-printed objects can inhibit corrosion behavior caused by surface defects. LPBF-AM-printed ships with superhydrophobic coating maintained great water repellency even after 10,000 cycles of seawater swashing. We believe that this study can provide a rapid and 3D fabrication solution for low-cost, highly efficient, and robust superhydrophobic coating, which is applicable to metal complex architectures toward corrosion-resistant requirements.

## Data Availability

Data are contained within the article and Supplementary Materials.

## Supplementary Materials

The following supporting information can be downloaded at: https://www.mdpi.com/article/10.3390/biomimetics8080598/s1, Figure S1: Optical images of LPBF-AM-printed samples with different laser powers and scanning speeds, resulting in different energy densities; Figure S2: The top surface height variation of LPBF-AM-printed samples with different energy densities along the diagonal line; Figure S3: Surface roughness (Sa) and surface morphologies of nine LPBF-AM-printed samples with different energy densities; Figure S4: The microstructures of LPBF-AM-printed samples with the optimal laser parameter under different abrasion conditions. (A) 1000 g, 50 abrasion cycles, (B) 1500 g, 50 abrasion cycles; Figure S5: Optical images and super depth-of-field images of LPBF-AM-printed samples with different groove widths before and after abrasion; Figure S6: Time-lapse images of water droplets bouncing on unabraded and abraded treated surfaces with different widths of grooves of 0.10 mm, 0.12 mm and 0.15 mm. Droplet sizes are about 4 mL; Figure S7: Micromorphology of powders and particles used in this work. (A) SEM image of stainless steel (316L) powder and its distribution of particle size. (B) SEM image of CoCr powder and its distribution of particle size. (C) SEM image of Ti powder and its distribution of particle size. (D) SEM image of NiTi powder and its distribution of particle size; Figure S8: Optical images and super depth-of-field images of samples with different materials before and after abrasion; Figure S9: Time-lapse images of water droplets bouncing on unabraded and abraded treated NiTi, CoCr, and Ti surfaces. Droplet sizes are about 4 mL; Figure S10: Optical images of mixing abrasion process progressively from knife cutting test, file underside and flank grinding test, to sandpaper friction test for LPBF-AM printed samples with different widths of grooves (0.10 mm, 0.12 mm, and 0.15 mm), and different material types (NiTi, CoCr, and Ti), as well as testing of superhydrophobicity after abrasion; Figure S11: SEM images of treated ship-like sample and untreated ship-like sample before and after 10,000 cycles swash. (A) before mixture washing and (B) 10,000 cycles of washing (scale bar 500 nm). Untreated ship-like sample (C) before mixture washing and (D) 10,000 cycles of washing (scale bar 400 μm); Table S1: Average contact angle, average sliding angle and roughness of LPBF-AM printed samples with different laser powers and scanning speeds; Movie S1: Linear abrasion testing of sample 3# (200 g, 5 kPa); Movie S2. Linear abrasion testing of sample 3# (500 g/12.5 kPa); Movie S3. Linear abrasion testing of sample 3# (1000 g/25 kPa); Movie S4. Linear abrasion testing of sample 3# (1500 g/37.5 kPa); Movie S5. Abrasion testing of samples with different widths of grooves; Movie S6. Time-lapse video of water droplets bouncing on unabraded and abraded treated surfaces with different widths of grooves of 0.10 mm, 0.12 mm, and 0.15 mm; Movie S7. Abrasion testing of samples with different materials; Movie S8. Time-lapse video of water droplets bouncing on unabraded and abraded treated NiTi, CoCr, and Ti surfaces; Movie S9. Demonstration of resistance of ship-like samples to simulated sea wave impact and corrosion.

## References

[1] Barthlott W., Neinhuis C. (1997). Purity of the sacred lotus, or escape from contamination in biological surfaces. Planta.

[2] Fürstner R., Barthlott W., Neinhuis C., Walzel P. (2005). Wetting and Self-Cleaning Properties of Artificial Superhydrophobic Surfaces. Langmuir.

[3] Mertaniemi H., Jokinen V., Sainiemi L., Franssila S., Marmur A., Ikkala O., Ras R.H. (2011). Superhydrophobic tracks for low-friction, guided transport of water droplets. Adv. Mater..

[4] Zang D., Zhu R., Zhang W., Yu X., Lin L., Guo X., Liu M., Jiang L. (2017). Corrosion-Resistant Superhydrophobic Coatings on Mg Alloy Surfaces Inspired by Lotus Seedpod. Adv. Funct. Mater..

[5] Wang L., Gong Q., Zhan S., Jiang L., Zheng Y. (2016). Robust Anti-Icing Performance of a Flexible Superhydrophobic Surface. Adv. Mater..

[6] Tian X., Verho T., Ras R.H.A. (2016). Moving superhydrophobic surfaces toward real-world applications. Science.

[7] Schutzius T.M., Jung S., Maitra T., Graeber G., Köhme M., Poulikakos D. (2015). Spontaneous droplet trampolining on rigid superhydrophobic surfaces. Nature.

[8] Verho T., Bower C., Andrew P., Franssila S., Ikkala O., Ras R.H.A. (2010). Mechanically Durable Superhydrophobic Surfaces. Adv. Mater..

[9] Lee K.-M., Park H., Kim J., Chun D.-M. (2019). Fabrication of a superhydrophobic surface using a fused deposition modeling (FDM) 3D printer with poly lactic acid (PLA) filament and dip coating with silica nanoparticles. Appl. Surf. Sci..

[10] Rather A.M., Manna U. (2016). Facile Synthesis of Tunable and Durable Bulk Superhydrophobic Material from Amine “Reactive” Polymeric Gel. Chem. Mater..

[11] Lu C., Gao Y., Yu S., Zhou H., Wang X., Li L. (2022). Non-Fluorinated Flexible Superhydrophobic Surface with Excellent Mechanical Durability and Self-Cleaning Performance. ACS Appl. Mater. Interfaces.

[12] Wang D., Sun Q., Hokkanen M.J., Zhang C., Lin F.-Y., Liu Q., Zhu S.-P., Zhou T., Chang Q., He B. (2020). Design of robust superhydrophobic surfaces. Nature.

[13] Dong Z., Cui H., Zhang H., Wang F., Zhan X., Mayer F., Nestler B., Wegener M., Levkin P.A. (2021). 3D printing of inherently nanoporous polymers via polymerization-induced phase separation. Nat. Commun..

[14] Mayer F., Ryklin D., Wacker I., Curticean R., Čalkovský M., Niemeyer A., Dong Z., Levkin P.A., Gerthsen D., Schröder R.R. (2020). 3D Two-Photon Microprinting of Nanoporous Architectures. Adv. Mater..

[15] Wu Z., Shi C., Chen A., Li Y., Chen S., Sun D., Wang C., Liu Z., Wang Q., Huang J. (2023). Large-Scale, Abrasion-Resistant, and Solvent-Free Superhydrophobic Objects Fabricated by a Selective Laser Sintering 3D Printing Strategy. Adv. Sci..

[16] Dong Z., Vuckovac M., Cui W., Zhou Q., Ras R.H.A., Levkin P.A. (2021). 3D Printing of Superhydrophobic Objects with Bulk Nanostructure. Adv. Mater..

[17] Lv X., Wang S., Xu Z., Liu X., Liu G., Cao F., Ma Y. (2023). Structural Mechanical Properties of 3D Printing Biomimetic Bone Replacement Materials. Biomimetics.

[18] Zhang L., Song B., Choi S.-K., Yao Y., Shi Y. (2022). Anisotropy-inspired, simulation-guided design and 3D printing of microlattice metamaterials with tailored mechanical-transport performances. Compos. Eng..

[19] Zhang L., Song B., Zhang J., Yao Y., Lu J., Shi Y. (2022). Decoupling microlattice metamaterial properties through a structural design strategy inspired by the Hall–Petch relation. Acta Mater..

[20] Zhang L., Song B., Yang L., Shi Y. (2020). Tailored mechanical response and mass transport characteristic of selective laser melted porous metallic biomaterials for bone scaffolds. Acta Biomater..

[21] Zhang L., Song B., Fu J., Wei S., Yang L., Yan C., Li H., Gao L., Shi Y. (2020). Topology-optimized lattice structures with simultaneously high stiffness and light weight fabricated by selective laser melting: Design, manufacturing and characterization. J. Manuf. Process..

[22] Cao L., Chen S., Wei M., Guo Q., Liang J., Liu C., Wang M. (2019). Effect of laser energy density on defects behavior of direct laser depositing 24CrNiMo alloy steel. Opt. Laser Technol..

[23] Raheem A.A., Hameed P., Whenish R., Elsen R.S., G A., Jaiswal A.K., Prashanth K.G., Manivasagam G. (2021). A Review on Development of Bio-Inspired Implants Using 3D Printing. Biomimetics.

[24] Maconachie T., Leary M., Lozanovski B., Zhang X., Qian M., Faruque O., Brandt M. (2019). SLM lattice structures: Properties, performance, applications and challenges. Mater. Des..

[25] Matthews R., Knutsen R., Westraadt J., Couvant T. (2021). Adaptation of the point defect model to simulate oxidation kinetics of 316L stainless steel in the pressurised water reactor environment. Corros. Sci..

[26] Hou B., Li X., Ma X., Du C., Zhang D., Zheng M., Xu W., Lu D., Ma F. (2017). The cost of corrosion in China. npj Mater. Degrad..

[27] González M., Saidman S. (2011). Electrodeposition of polypyrrole on 316L stainless steel for corrosion prevention. Corros. Sci..

[28] Xiang T., Han Y., Guo Z., Wang R., Zheng S., Li S., Li C., Dai X. (2018). Fabrication of Inherent Anticorrosion Superhydrophobic Surfaces on Metals. ACS Sustain. Chem. Eng..

[29] Lu Y., Sathasivam S., Song J., Crick C.R., Carmalt C.J., Parkin I.P. (2015). Repellent materials. Robust self-cleaning surfaces that function when exposed to either air or oil. Science.

[30] Velayi E., Norouzbeigi R. (2017). Robust superhydrophobic needle-like nanostructured ZnO surfaces prepared without post chemical-treatment. Appl. Surf. Sci..

[31] Peng J., Zhao X., Wang W., Gong X. (2019). Durable Self-Cleaning Surfaces with Superhydrophobic and Highly Oleophobic Properties. Langmuir.

[32] Zhou H., Chen R., Liu Q., Liu J., Yu J., Wang C., Zhang M., Liu P., Wang J. (2019). Fabrication of ZnO/epoxy resin superhydrophobic coating on AZ31 magnesium alloy. Chem. Eng. J..

[33] Hou K., Zeng Y., Zhou C., Chen J., Wen X., Xu S., Cheng J., Pi P. (2018). Facile generation of robust POSS-based superhydrophobic fabrics via thiol-ene click chemistry. Chem. Eng. J..

[34] Gao J., Luo J., Wang L., Huang X., Wang H., Song X., Hu M., Tang L.-C., Xue H. (2019). Flexible, superhydrophobic and highly conductive composite based on non-woven polypropylene fabric for electromagnetic interference shielding. Chem. Eng. J..

[35] Wang N., Xiong D., Deng Y., Shi Y., Wang K. (2015). Mechanically Robust Superhydrophobic Steel Surface with Anti-Icing, UV-Durability, and Corrosion Resistance Properties. ACS Appl. Mater. Interfaces.

[36] Gao X., Zhou J., Du R., Xie Z., Deng S., Liu R., Liu Z., Zhang J. (2016). Robust Superhydrophobic Foam: A Graphdiyne-Based Hierarchical Architecture for Oil/Water Separation. Adv. Mater..

[37] Zhou C., Chen Z., Yang H., Hou K., Zeng X., Zheng Y., Cheng J. (2017). Nature-Inspired Strategy toward Superhydrophobic Fabrics for Versatile Oil/Water Separation. ACS Appl. Mater. Interfaces.

[38] Shen L., Xu M., Jiang W., Qiu M., Fan M., Ji G., Tian Z. (2019). A novel superhydrophobic Ni/Nip coating fabricated by magnetic field induced selective scanning electrodeposition. Appl. Surf. Sci..

[39] Vazirinasab E., Jafari R., Momen G. (2018). Application of superhydrophobic coatings as a corrosion barrier: A review. Surf. Coat. Technol..

[40] Davies J., Truong-Ba H., Cholette M.E., Will G. (2021). Optimal inspections and maintenance planning for anti-corrosion coating failure on ships using non-homogeneous Poisson Processes. Ocean Eng..

[41] Zhang H., Yan L., Zhu Y., Ai F., Li H., Li Y., Jiang Z. (2021). The Effect of Immersion Corrosion Time on Electrochemical Corrosion Behavior and the Corrosion Mechanism of EH47 Ship Steel in Seawater. Metals.

