# Supplementary material for: Robust Superhydrophobicity through Surface Defects from Laser Powder Bed Fusion Additive Manufacturing
Source: Biomimetics (Basel). 2023 Dec 12;8(8):598. doi: 10.3390/biomimetics8080598 (PMC10741415; doi:10.3390/biomimetics8080598)
Supplement: Supplementary file 1 [file biomimetics-08-00598-s001.zip › biomimetics-2690077-supplementary.pdf]

Supplementary Materials

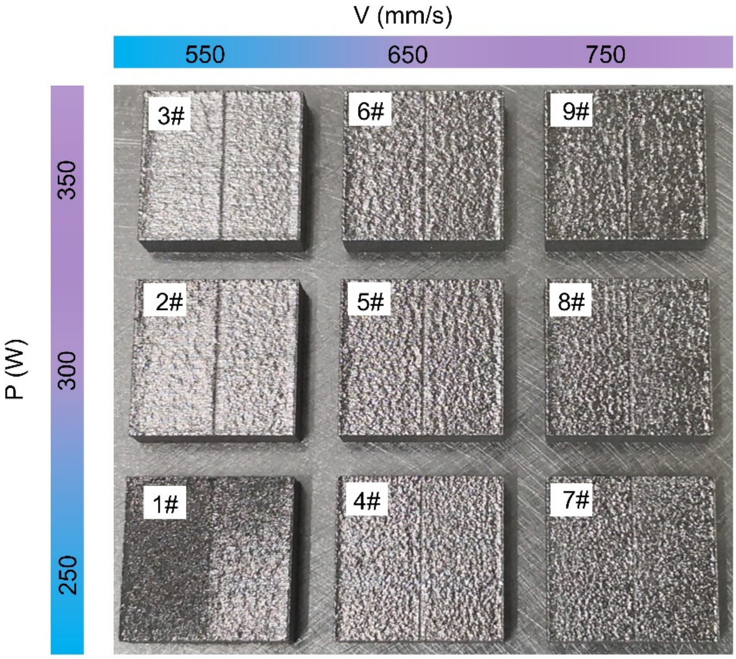

Fig. S1. Optical images of LPBF-AM printed samples with different laser powers and scanning speeds, resulting in different energy densities.

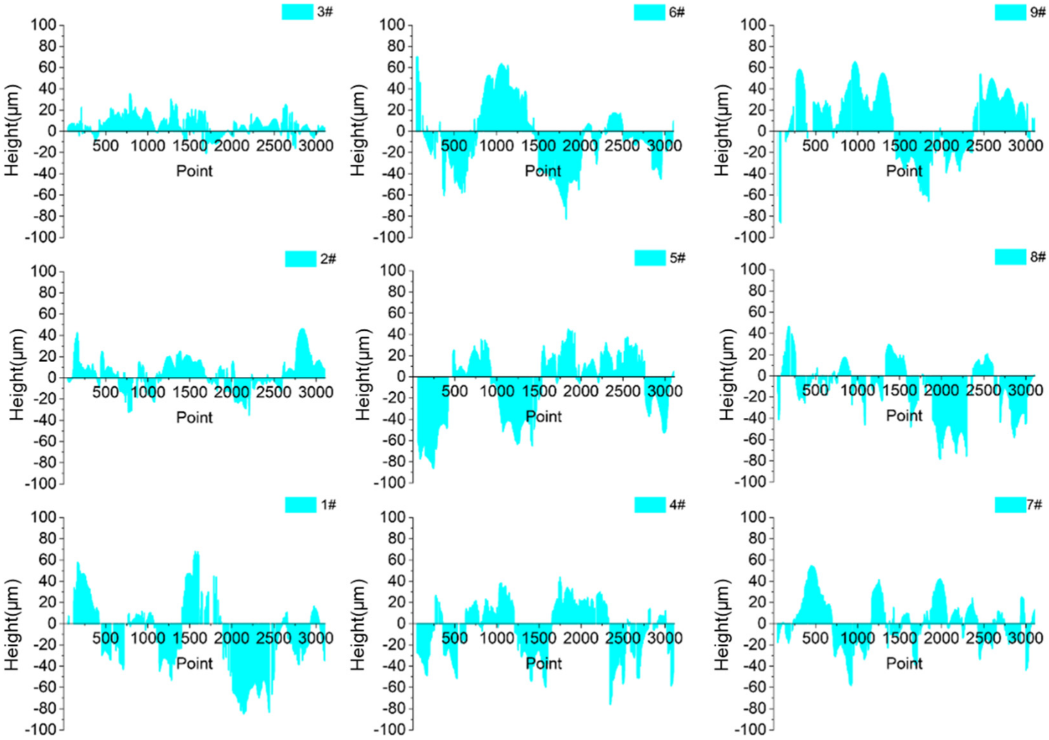

Fig. S2. The top surface height variation of LPBF-AM printed samples with different energy densities along the diagonal line.

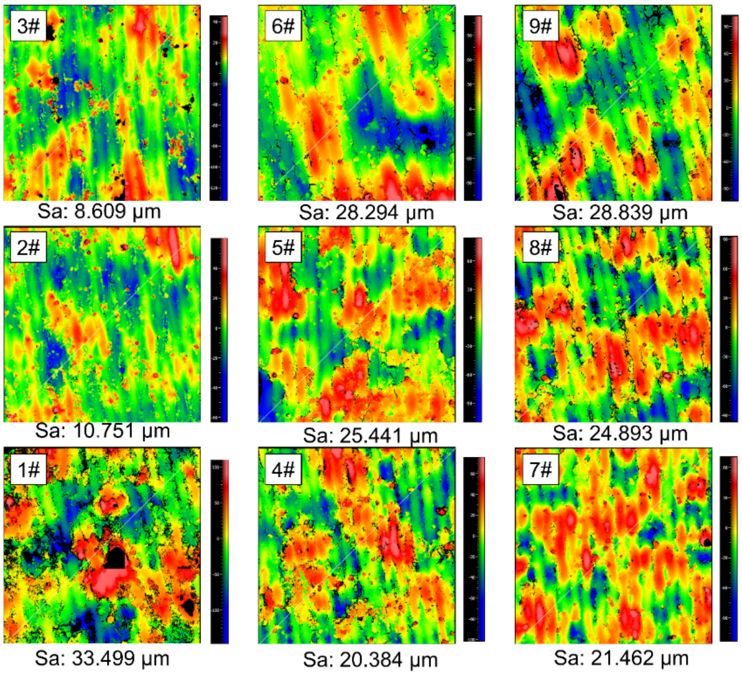

Fig. S3. Surface roughness (Sa) and surface morphologies of nine LPBF-AM printed samples with different energy densities.

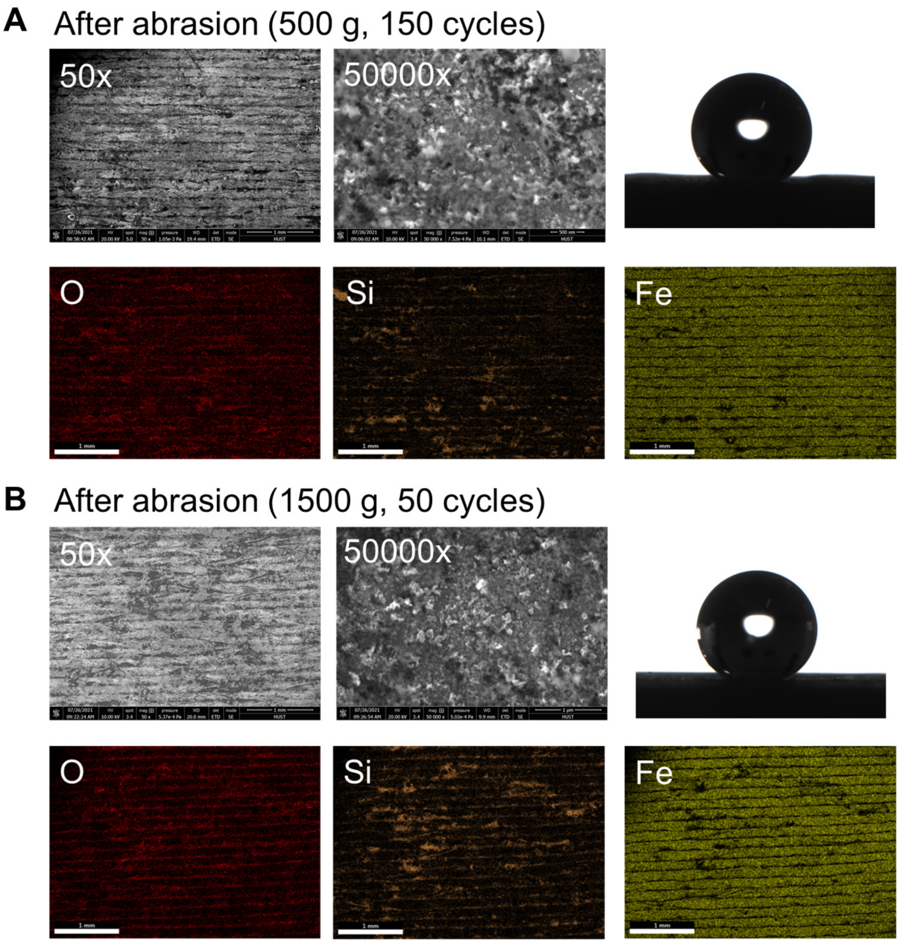

Fig. S4. The microstructures of LPBF-AM printed samples with the optimal laser parameter under different abrasion conditions. A) 1000g, 50 abrasion cycles, B) 1500g, 50 abrasion cycles.

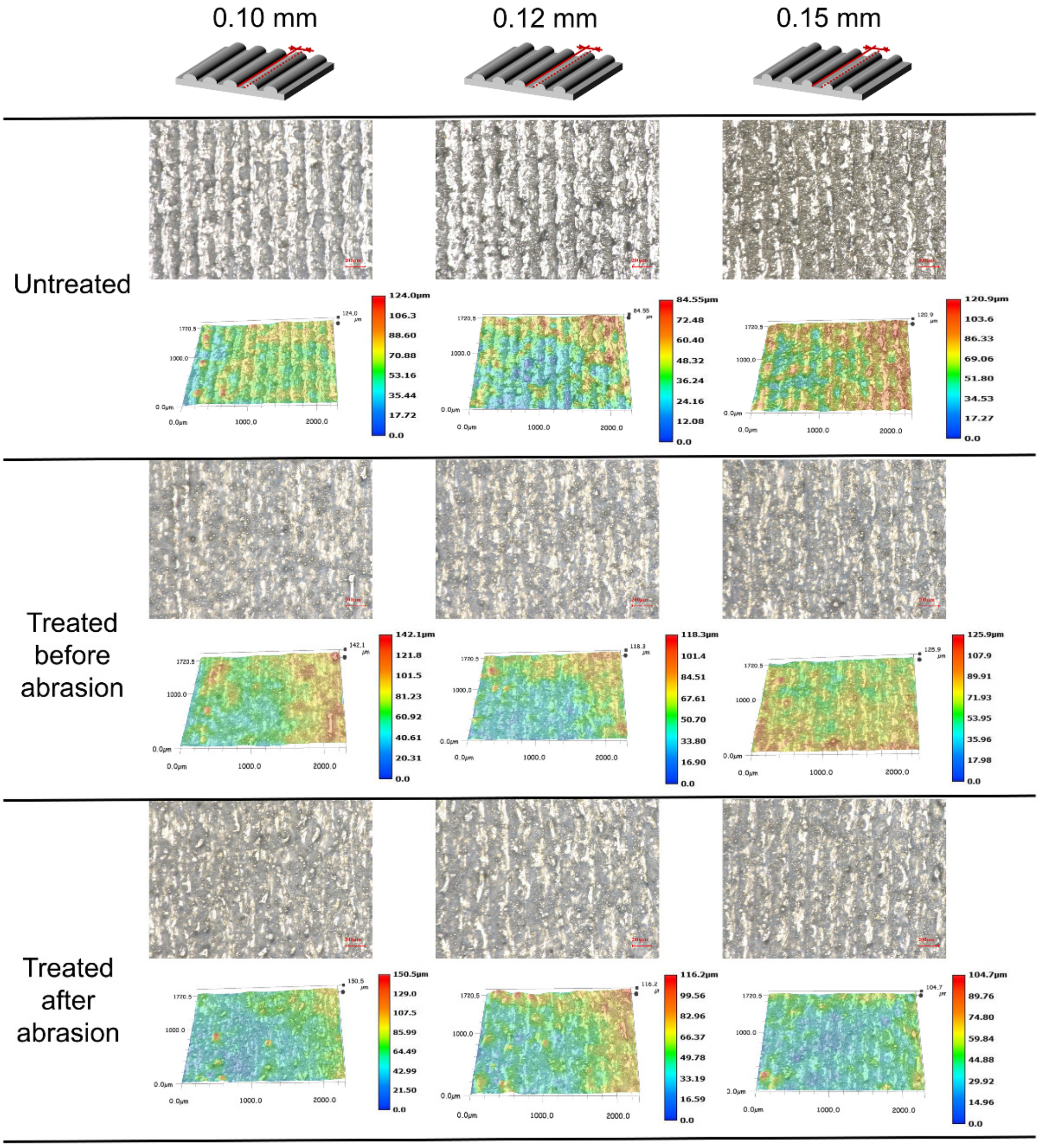

Fig. S5. Optical images and super depth-of-field images of LPBF-AM printed samples with different groove widths before and after abrasion.

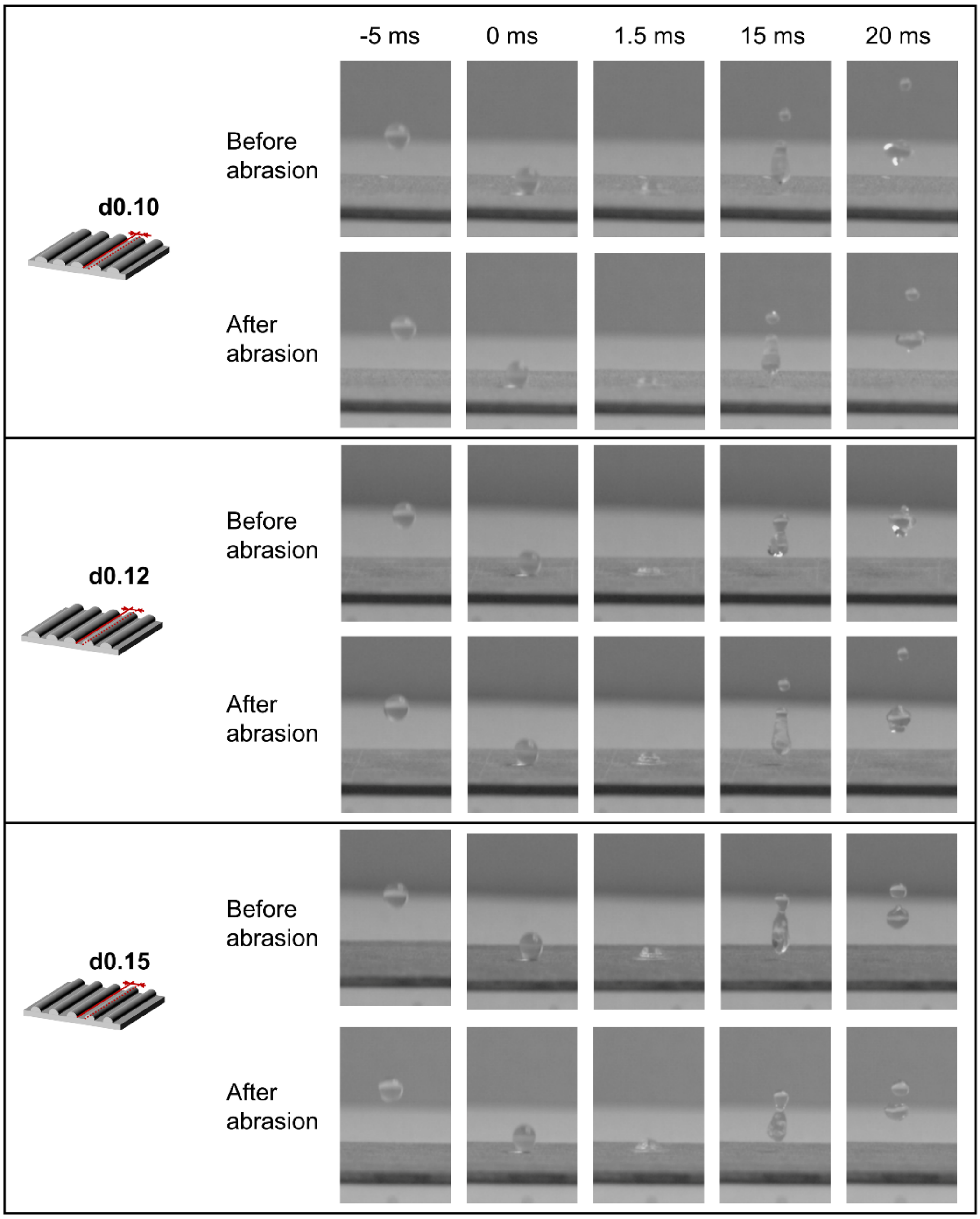

Fig. S6. Time-lapse images of water droplets bouncing on unabraded and abraded treated surfaces with different widths of grooves of 0.10 mm, 0.12 mm and 0.15 mm. Droplet sizes are about 4 mL.

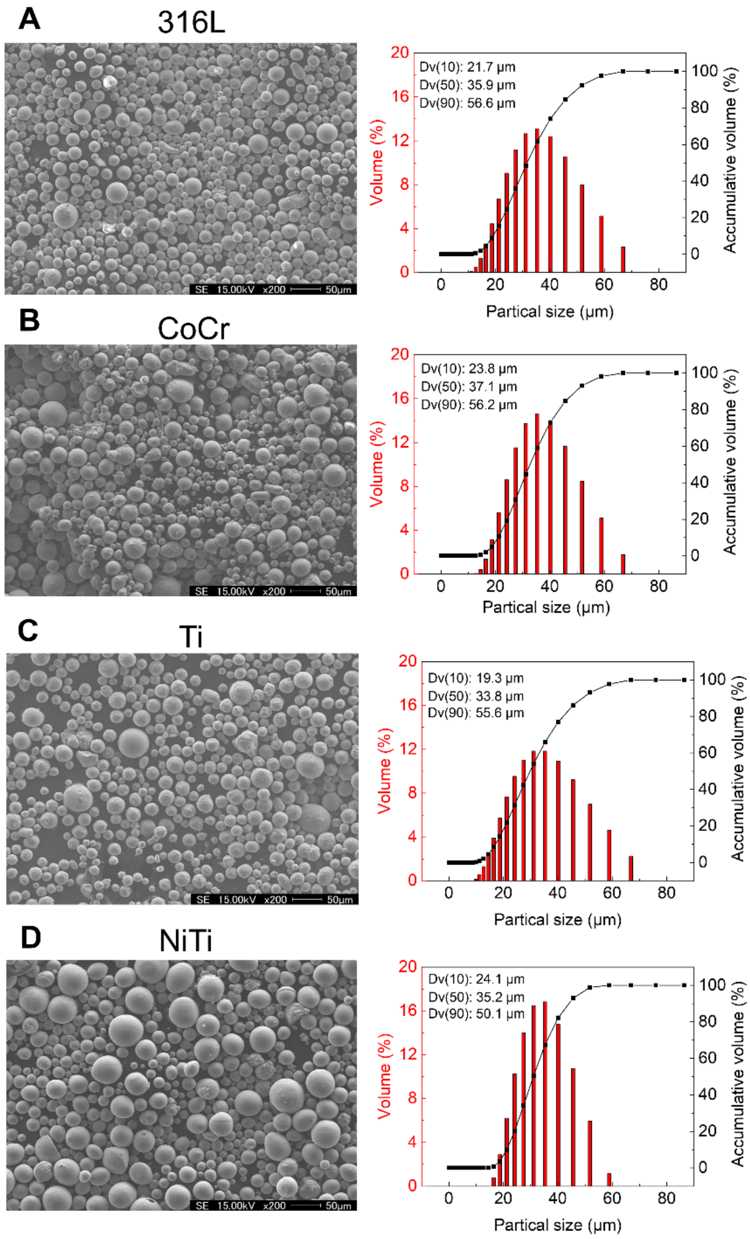

Fig. S7. Micromorphology of powders and particles used in this work. A) SEM image of stainless steel (316L) powder and its distribution of particle size. B) SEM image of CoCr powder and its distribution of particle size. C) SEM image of Ti powder and its distribution of particle size. D) SEM image of NiTi powder and its distribution of particle size.

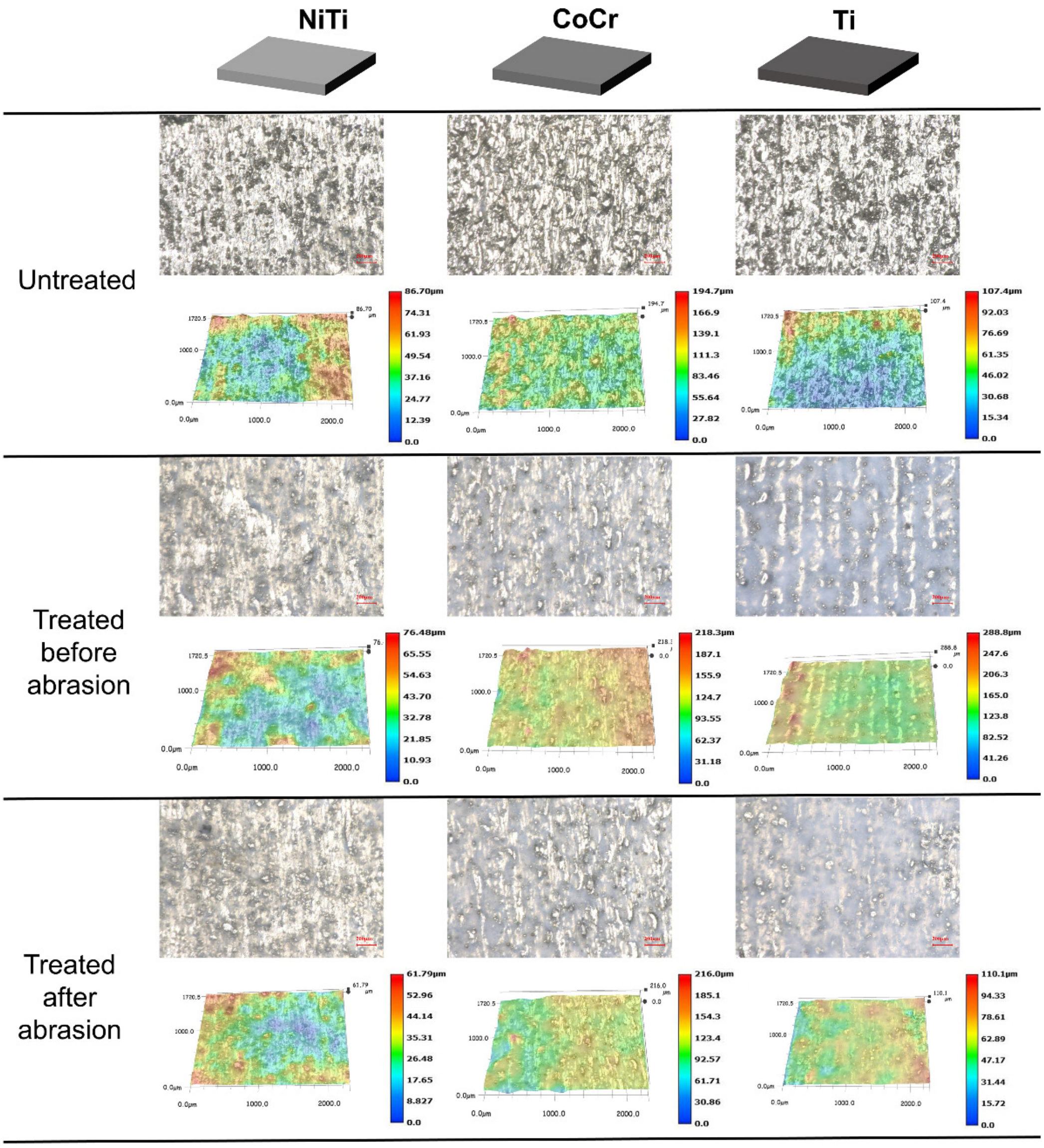

Fig. S8. Optical images and super depth-of-field images of samples with different materials before and after abrasion.

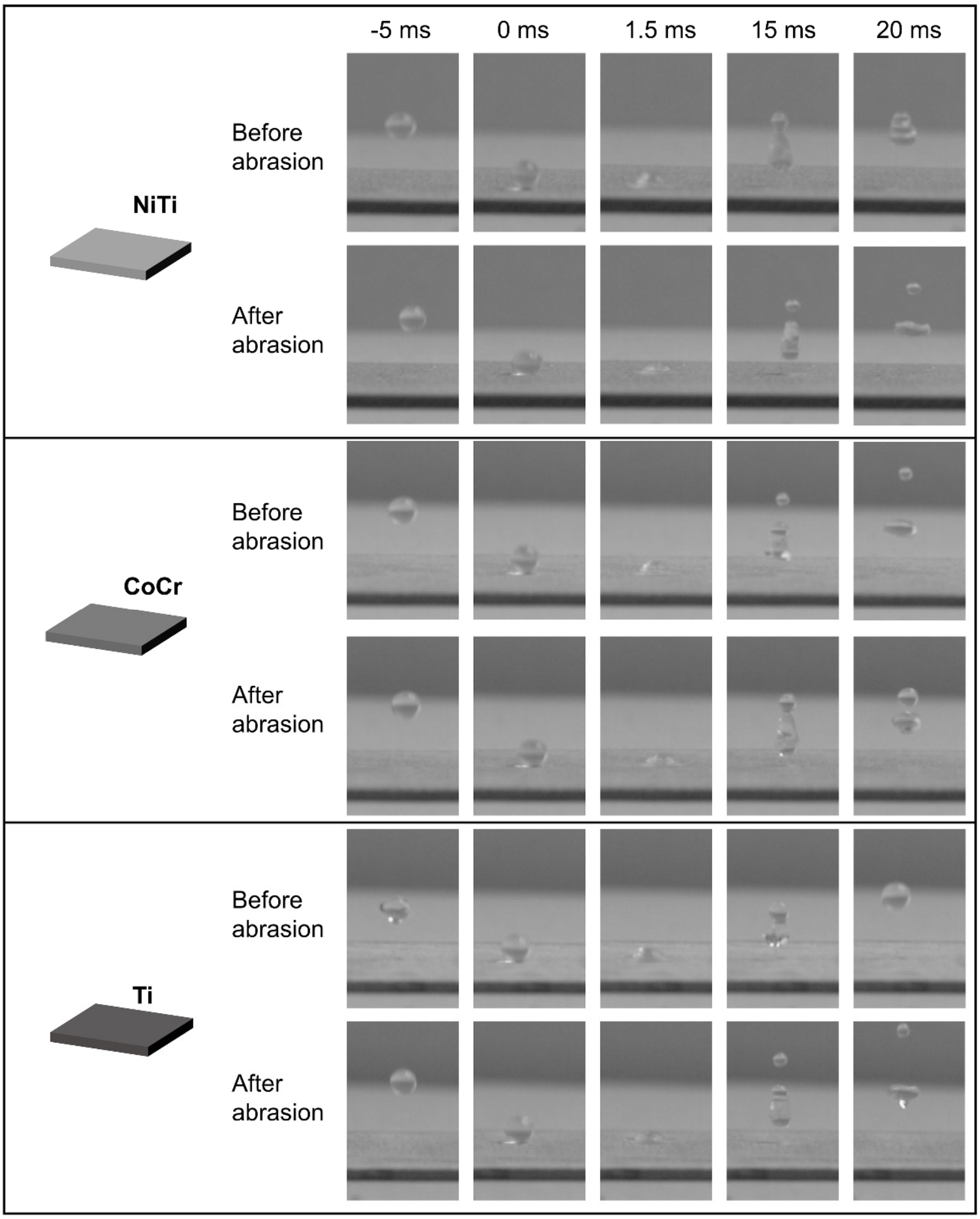

Fig. S9. Time-lapse images of water droplets bouncing on unabraded and abraded treated NiTi, CoCr and Ti surfaces. Droplet sizes are about 4 mL.

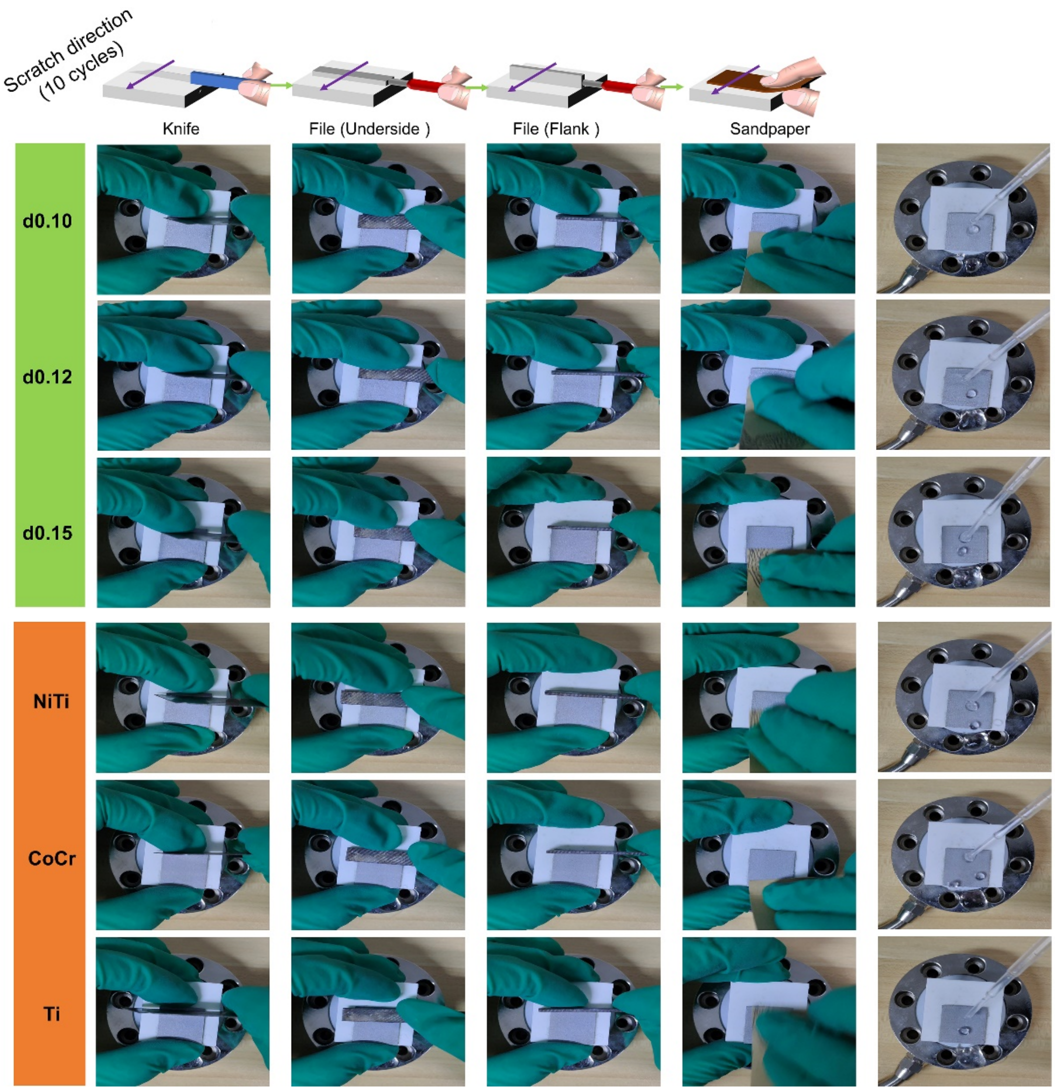

Fig. S10. Optical images of mixing abrasion process progressively from knife cutting test, file underside and flank grinding test, to sandpaper friction test for LPBF-AM printed samples with different widths of grooves (0.10 mm, 0.12 mm and 0.15 mm), and different material types (NiTi, CoCr, and Ti), as well as testing of superhydrophobicity after abrasion.

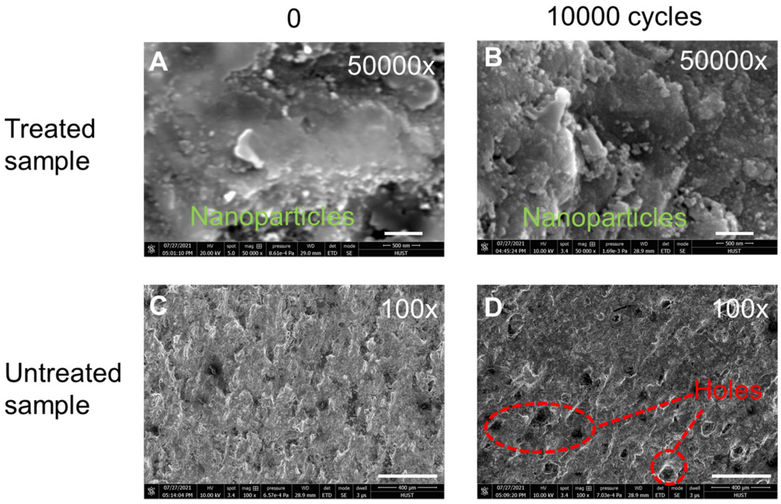

Fig. S11. SEM images of treated ship-like sample and untreated ship-like sample before and after 10,000 cycles swash. A) before mixture washing and B) 10,000 cycles of washing (scale bar 500 nm). Untreated ship-like sample C) before mixture washing and D) 10,000 cycles of washing (scale bar 400 μm).

Table S1 Average contact angle, average sliding angle and roughness of LPBF-AM printed samples with different laser powers and scanning speeds.

Sample number	Average contact angle (°)	Average sliding angle (°)	Roughness Sa (μm)
1#	143.8	28.6	33.499
2#	145.6	22.7	10.751
3#	150.0	10.4	8.609
4#	148.5	21.9	20.384
5#	146.7	16.3	25.441
6#	144.0	23.4	28.294
7#	149.2	26.2	21.462
8#	145.4	18.5	24.893
9#	144.5	31.8	28.839

Movie S1. Linear abrasion testing of sample 3# (200g, 5kPa).

Movie S2. Linear abrasion testing of sample 3# (500g/12.5kPa).

Movie S3. Linear abrasion testing of sample 3# (1000g/25kPa).

Movie S4. Linear abrasion testing of sample 3# (1500g/37.5kPa).

Movie S5. Abrasion testing of samples with different widths of grooves.

Movie S6. Time-lapse video of water droplets bouncing on unabraded and abraded treated surfaces with different widths of grooves of 0.10 mm, 0.12 mm, and 0.15 mm.

Movie S7. Abrasion testing of samples with different materials.

Movie S8. Time-lapse video of water droplets bouncing on unabraded and abraded treated NiTi, CoCr and Ti surfaces.

Movie S9. Demonstration of resistance of ship-like samples to simulated sea wave impact and corrosion.
